# Modeling Colloidal Particle Aggregation Using Cluster Aggregation with Multiple Particle Interactions

**DOI:** 10.1021/acs.jpcb.3c07992

**Published:** 2024-04-30

**Authors:** Jakob Antonsson, Charlotte Hamngren Blomqvist, Eva Olsson, Tobias Gebäck, Aila Särkkä

**Affiliations:** † Department of Mathematical Sciences, Chalmers University of Technology and University of Gothenburg, SE-412 96 Gothenburg, Sweden; ‡ Department of Physics, Chalmers University of Technology, SE-412 96 Gothenburg, Sweden

## Abstract

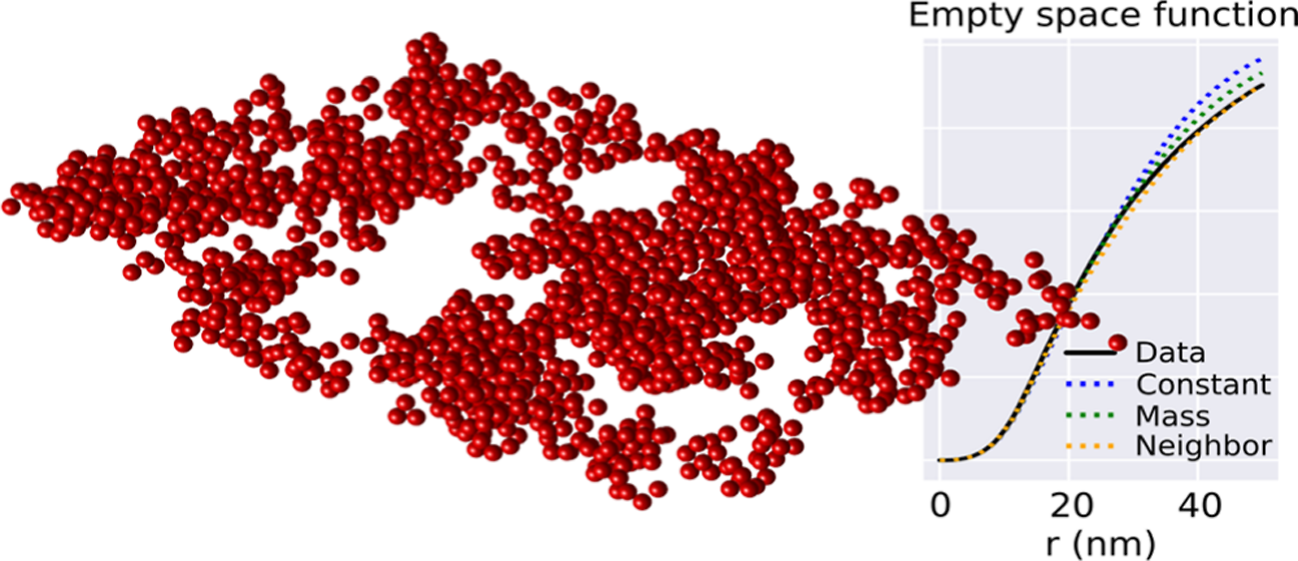

In this study, we investigate the aggregation dynamics of colloidal silica by generating simulated structures and comparing them to experimental data gathered through scanning transmission electron microscopy (STEM). More specifically, diffusion-limited cluster aggregation and reaction-limited cluster aggregation models with different functions for the probability of particles sticking upon contact were used. Aside from using a constant sticking probability, the sticking probability was allowed to depend on the masses of the colliding clusters and on the number of particles close to the collision between clusters. The different models of the sticking probability were evaluated based on the goodness-of-fit of spatial summary statistics. Furthermore, the models were compared to the experimental data by calculating the structures’ fractal dimension and mass transport properties from simulations of flow and diffusion. The sticking probability, depending on the interaction with multiple particles close to the collision site, led to structures most similar to the STEM data.

## Introduction

Irreversible colloidal aggregation is relevant for various industrial applications, for example, in food science, battery science, and medicine.^1−3^ More specifically, it is important to maintain the stability of the system in biological fluids such as blood and milk. Targeted drug delivery systems or diagnostic systems for protein aggregation-related neurodegenerative diseases are examples of medical applications, where prediction of colloidal aggregation is crucial.^4^ Even for water purification or soil amelioration, aggregation together with sedimentation can be of interest.^5^ Colloidal aggregation is also relevant in the design of new materials. Often, specific mechanical or mass transport properties are sought, and research has been carried out to understand and predict the aggregation dynamics during the formation of such materials.

A wide range of different structures can be obtained for silica nanoparticle gels, and therefore, they are of great interest for studying colloidal aggregation.^6^ A stable colloidal suspension (hydrosol) is maintained if the electromagnetic repulsion between particles is strong enough to hinder aggregation. Colloidal silica nanoparticles have a negatively charged surface layer, the so-called Stern layer,^7^ which can be shielded with a diffuse electrostatic layer attributed to the solute.^8^ An added excess of cations is attracted to the negative surfaces of the silica particles, thereby reducing their surface charge. Consequently, the repulsive electromagnetic forces are diminished, and at a critical concentration of cations, the formation of a silica hydrogel starts. The concentration of cations, as well as the particle size, concentration, solute type and temperature, impacts both how fast the gel is formed and its final structure in a fairly complex way.^8^ The practical dynamics of the aggregation process have earlier been investigated with different light-scattering measurements.^9,10^

Since the physicochemical forces during the aggregation of silica are complex, simplified mathematical models have been proposed. One of the more prominent models is the Derjaguin–Landau–Verwey–Overbeek (DLVO) theory, in which the interaction between particles is assumed to consist of electrostatic repulsion and London-van der Waals attraction.^11^ The DLVO theory is also used to describe the interaction potential of the morphological aggregation model, which uses Lagrangian simulations as the reference system. The model has been suggested to simulate single aggregates with a given fractal dimension and number of elementary objects.^12^ Simulations of gel formation using DLVO theory potentials are possible using dissipative particle dynamics^13,14^ or the discrete element method (DEM) using Langevin dynamics to model diffusion.^15,16^ For example, it was shown using DEM simulations in 2D that an increased Debye length raised the potential barrier between particles, thus preventing aggregation from occurring.^15^ It is also possible to include other effects such as shear flows and rotation of particles with heterogeneous surfaces.^17,18^ However, for silica, experiments have shown that other short-range forces play an important role in the interaction process.^19^ In addition, the DLVO theory only considers the interaction between pairs of single particles and not clusters, and it has therefore been difficult to satisfactorily explain the behavior of colloidal silica using the DLVO theory solely.^11,20^

An alternative model for aggregation, or more specifically for the time evolution of the cluster size distribution, was suggested by Smoluchowski.^21^ It is a mean field model where a single cluster of particles is embedded in a soup of other clusters. The time evolution in the Smoluchowski model is simulated using the so-called coagulation kernel, which describes the rate at which particles and clusters coagulate. This kernel typically includes probabilities for the aggregation between particles and clusters. This theory was further modified by incorporating hydrodynamic resistances into the interparticle interaction force in addition to electrostatic repulsion and van der Waals attraction.^22^ However, even the modified model fails to describe the aggregation kinetics for small nanosilica particles with diameters less than 190 nm.^23^

On the atomic scale, molecular dynamics models have been used to investigate the internal structure of silica nanoparticles,^24^ as well as to study the binding and subsequent ripening of bound silica nanoparticles.^25−27^ In addition, they have been used to study the structural properties of such nanoparticles via the radial distribution function, mean squared displacement, coordination numbers, and bond-angle distributions.^28^ The normal contact and noncontact forces between two silica nanoparticles in a Lennard-Jones liquid can also be calculated by using molecular dynamics.^29^ Furthermore, so-called multiparticle collision dynamics or stochastic rotation dynamics method, which alternates between streaming and collision steps in an ensemble of point particles, has been studied.^30^ While providing detailed knowledge on the interaction between pairs of particles, these methods are too costly to be used to simulate gel formation with a large number of nanoparticles due to the large number of atoms involved. A simple molecular dynamics simulation of a small system can take anywhere from a few minutes to several hours, and a large, more complex system can take days or even weeks to run on an average personal computer. Therefore, molecular dynamics simulations are typically performed on computer clusters or supercomputers using several processors in parallel.^31^

A common approach to model the gel formation is to use diffusion-limited cluster aggregation (DLCA) and reaction-limited cluster aggregation (RLCA), where nanoparticles are treated as spheres undergoing Brownian motion and the complex interaction between pairs of particles is reduced to a probability of aggregation upon collision.^32−34^ Despite their simplicity, these models are able to produce gel structures that are similar to experimental ones, as shown e.g. by earlier work in our group^35^ and studies where DLCA models are used to study the structural and mechanical properties of silica aerogels.^36^ The probability of aggregation in the DLCA model equals one, while it is less than one but fixed in the RLCA model. Typically, in dynamic cluster aggregation simulations, the probability of bonds forming between colliding clusters, often termed the sticking probability, remains constant for all collisions. However, efforts have been made to introduce dependencies into these probabilities. One approach is to make the sticking probability dependent on the sizes of the colliding clusters. This adjustment can be interpreted as a first approximation of long-range particle–particle interaction between the clusters.^37^ Nevertheless, the cluster sizes may also be correlated to other factors that influence the aggregation dynamics.

In this work, we apply and extend the RLCA model. Our aim is to provide an efficient method that reproduces the structure of the final silica gel accurately rather than to understand the detailed physics of the aggregation process, which makes the RLCA model an appropriate choice. Structures generated by dynamic three-dimensional models of the aggregation are compared to experimental data from a silica nanoparticle gel acquired by using scanning transmission electron microscopy (STEM). In the earlier study by our group,^35^ the same data set as the one used in this paper was studied and compared to structures formed through DLCA and RLCA simulations. The generated structures agreed rather well, but not perfectly, with the microscopy data. This study (which is mainly based on the Master’s thesis by the first author) is an extension of our earlier work.^35^ In addition to using only a constant probability of binding under collision, two other models were investigated in this paper. In the first one, the probability was made to depend on the masses of the colliding clusters, and in the other, it was made to depend on the number of particles close to the collision. Furthermore, in the simulations in study by Häbel et al.,^35^ all primary particles had a constant diameter, but they noticed variations in the diameter in the experimental gel structure. Here, DLCA and RLCA simulations were instead performed with particles with diameters sampled from a distribution fitted to the experimental data.

The goal of this study is to develop a method for generating structures as similar as possible to real silica structures. By comparison of the computer generated structures with the experimental STEM data, insights into the aggregation dynamics can also be gained. Part of the comparison is based on using spatial summary statistics from point process theory, in which the particle centroids are considered as realizations of point processes. These summary statistics give important information about the spatial structure of the gel, such as the amount of empty space and the shape of local clusters. However, to the best of our knowledge, they have earlier been used in colloidal aggregation studies only by us.^35,38^ In addition, the fractal dimensions and mass transport properties of the simulated and experimental structures are compared.

The results show an improved fit of the generated structures to the microscopy data with the chosen methods of evaluation when the number of particles close to the colliding pair influences the probability of aggregation. The main conclusion is, therefore, that the proposed method generates more realistic structures than standard RLCA. Furthermore, the results indicate that the forces acting between silica particles may be influenced by the neighboring particles.

## Materials and Methods

### Experimental Data Acquisition

#### Aggregated Silica Particle Gel Production

An aggregated silica particle hydrogel was formed from a filtered 9 wt % (4.1 vol %) colloidal suspension of amorphous silica nanoparticles (Bindzil 40/130; AkzoNobel, PPC AB, Bohus, Sweden).^39,40^ In short, the gelation process was triggered by adjusting the pH from 9.1 to 7.8 and by adding NaCl (s) to a final concentration of 0.5 M NaCl (aq). The gel was left to set for 2 weeks before electron microscopy preparation.

#### Sample Preparation and Electron Microscopy Data Acquisition

Cubes with the side of 1 mm were cut from the gel, dehydrated in a graded ethanol and propylene oxide series, infiltrated with TLV resin (TAAB Laboratories, Berks, England), embedded in a TLV resin stub, and polymerized at 60 °C overnight. The sample was sectioned to a thickness of 200 nm and transferred to a 200 mesh Cu grid.

A high-angle annular dark field STEM (HAADF STEM) tomogram of a silica gel section was acquired using an FEI Titan 80–300 equipped with a field electron gun (FEI Company, Eindhoven, Netherlands), operated at 300 kV.^41^ The sample was imaged at 1° inclination intervals between 70° and −74°. Alignment and reconstruction of the tomogram using the simultaneous iterative reconstruction technique^42^ with 30 iterations and four times binning were performed in Inspect3D (FEI Company). Further details on sample production, preparation, and tomography are found in earlier work.^39,43,44^

**Figure 1:**
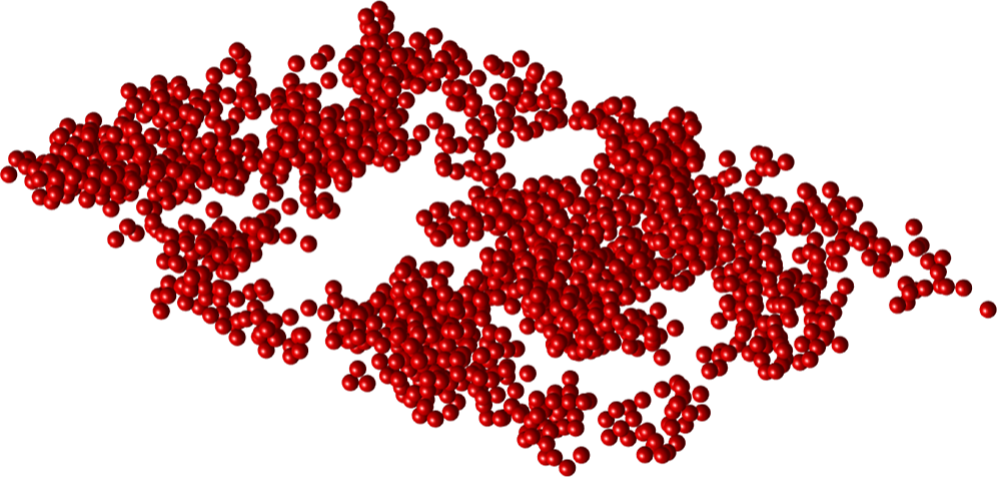
Geometrical representation of the STEM data where all particles have been assumed to have a diameter of 21 nm.

From the reconstructed tomogram (with a voxel size of 1.44 nm^3^), a final stack of two-dimensional grayscale images was extracted. This extracted subset for statistical analysis has a volume of 740 × 1075 × 100 nm^3^. As previously described,^35,43^ the data were filtered, equalized, and segmented into a silica or void phase creating a binary data set. The reconstructed tomogram was then masked by this binary data set to identify the center points of the silica particles in the 3D volume, which are illustrated in Figure 1.

#### Estimation of Particle Size Distribution

**Figure 2:**
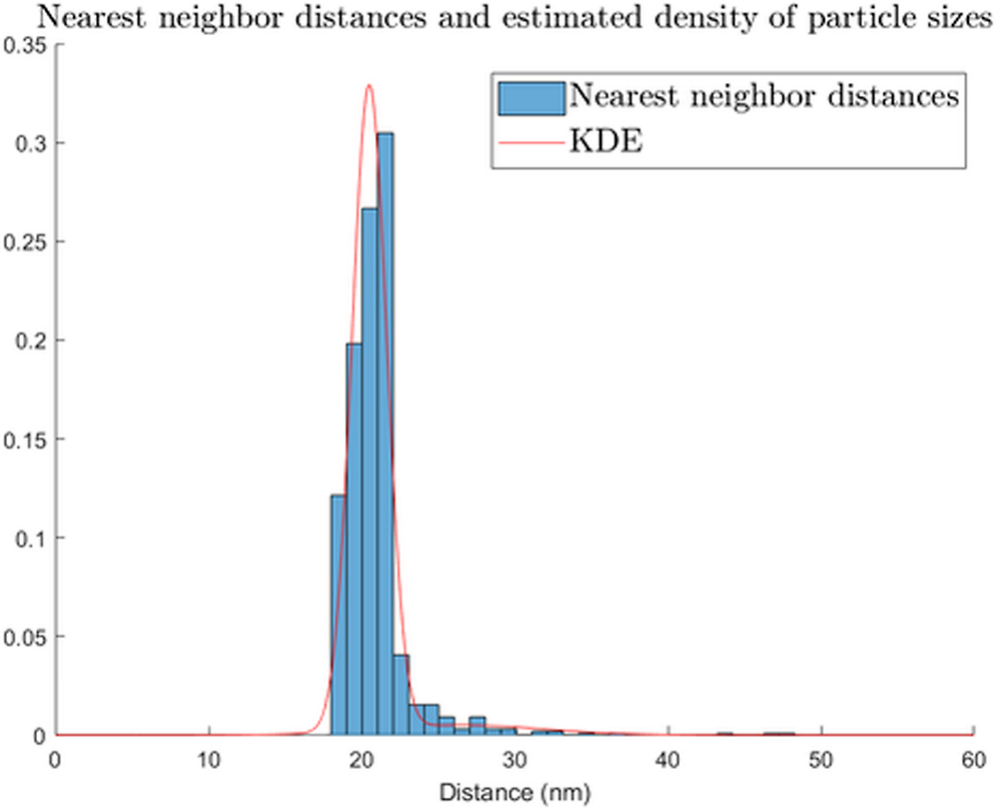
Histogram of nearest neighbor distances from the experimental data and the estimated probability density function of particle diameters.

The distribution of particle sizes was estimated from the nearest neighbor distances in the STEM data, *d*_1_, ..., *d*_*n*_ for the *n* particles by using kernel density estimation (KDE)
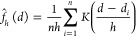
1Here, *K*(·) is the kernel smoothing function and *h* is the bandwidth. The standard normal density function was chosen as smoothing function *K*(·), and the bandwidth was selected using Silverman’s rule of thumb
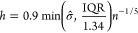
2where σ̂ is the sample standard deviation and IQR is the interquartile range of the data.^45^ A histogram of the experimental data can be seen along with the estimated density in Figure 2. The expected value of the nearest neighbor distance was found to be 21 nm, which we interpret as the mean particle diameter.

### Aggregation Simulation Models

#### DLCA and RLCA

DLCA and RLCA are two different regimes of irreversible cluster aggregation.^32^ In DLCA, repulsion between particles is negligible, whereas, in RLCA, strong repulsive forces between particles make it more difficult for bonds to be formed. Cluster aggregation in these regimes was simulated by letting particles move according to Brownian motion. When clusters collide, a probability *p* was calculated for the colliding particles to bind to each other. This probability *p* will be called the sticking probability, and the DLCA and RLCA regimes correspond to *p* = 1 and *p* ≪ 1, respectively.

#### Models for Sticking Probability

The cluster aggregation simulations were carried out with three different functions for the sticking probability. In the first model, the sticking probability was constant throughout the simulations, providing us with a good baseline to evaluate the other models.

The second model that was investigated was made dependent on the masses of the colliding clusters. Similar probability models have been used in several previous aggregation studies, such as those conducted by Family et al.^46^ and Li and Xiong.^47^ Here, we considered a sticking probability function of the form
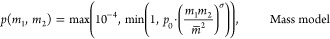
3where *p*_0_ ∈ [0, 1] and  are model parameters to be estimated, *m̅* is the average particle mass, and *m*_1_ and *m*_2_ are the masses of the colliding clusters. Hence, collisions between single particles with average mass have a sticking probability of *p*_0_. As clusters grow larger, this probability will increase with the masses of the clusters if σ > 0 and decrease if σ < 0. Here, a lower limit of 10^–4^ for *p*(*m*_1_, *m*_2_) was introduced to limit the computational time. This particular lower limit seems reasonable since the aggregation probabilities 10^–3^ and 10^–4^ tend to result in very similar structures.^35^

**Figure 3:**
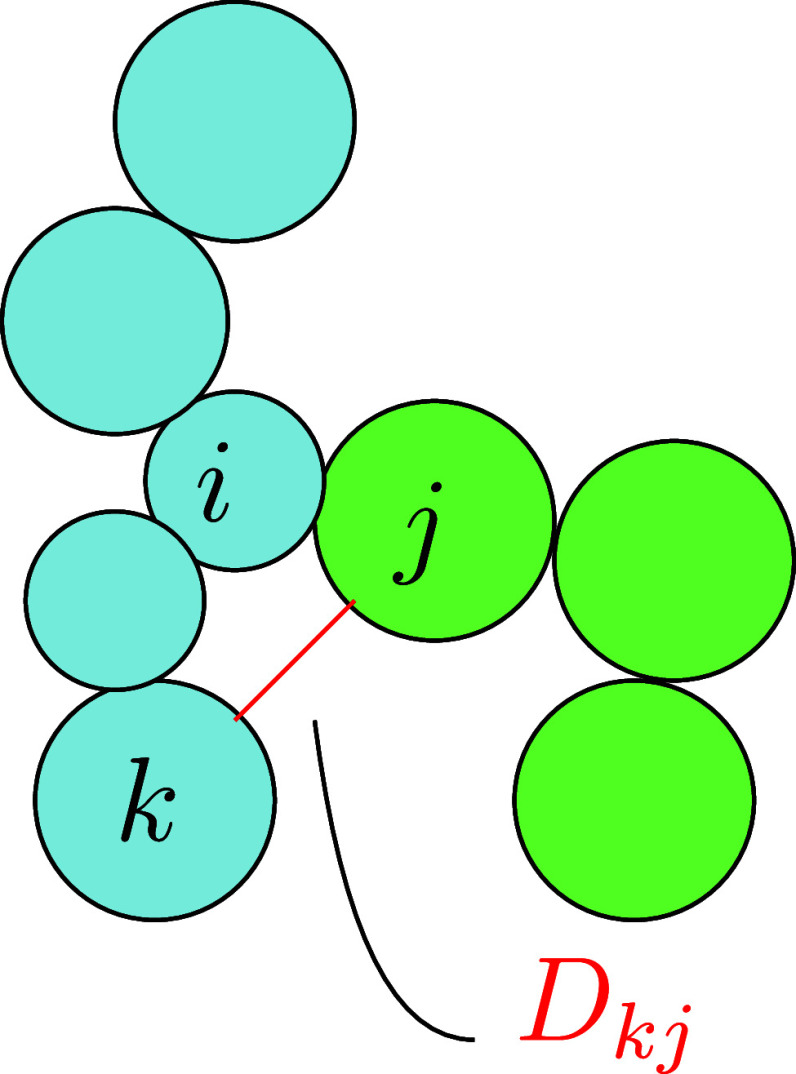
Example of how the sticking probability in eq 4 is calculated. If the distance *D*_*kj*_ between the surfaces of one of the colliding particles *j* and a particle in the opposite cluster *k* (which is not the particle *i* that *j* has collided with) is less than *s* + *r*_*k*_ + *r*_*j*_, particle *k* contributes with δ to the sticking probability. The corresponding calculation is carried out for all particles in an opposite cluster to the colliding particles.

Finally, in the third model, the sticking probability was made dependent on the number of particles close to the collision. More specifically, let *C*_1_ and *C*_2_ denote two colliding clusters and let *D*_*ij*_ be the distance between the surfaces of two particles *i* and *j* with radii *r*_*i*_ and *r*_*j*_. The sticking probability for a collision between particles *i* ∈ *C*_1_ and *j* ∈ *C*_2_ can then be written as
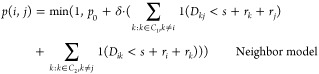
4where *p*_0_ ∈ [0, 1] and δ, *s* ≥ 0 are model parameters. The minimum is taken since the probability can be at most 1. All collisions therefore have a probability of at least *p*_0_. The probability then increases with δ for every other particle in an opposite cluster within surface-to-surface distance *s* + *r*_*i*_ + *r*_*k*_ of the colliding ones. Here, *r*_*i*_ and *r*_*k*_ are the radii of one of the colliding particles and the neighbor to which the distance is measured (see Figure 3). The sticking probability can be interpreted to take into account multiple particle interactions at the collision site, where larger particles interact over a greater distance.

#### Implementation

The aggregation simulations were carried out by using an in-house Fortran code. The simulation box was set to be 700 × 700 × 700 nm,^3^ containing 7326 nanoparticles with diameters sampled from the distribution fitted to the experimental data. Assuming a fixed particle diameter of 21 nm, this results in a solid volume fraction of ϕ = 0.104. However, because of the size distribution, the solid volume fraction varied between the generated structures. Diffusion of the particles was simulated using periodic boundary conditions until all particles were connected, resulting in one big cluster. The diffusivity of clusters was made dependent on cluster mass *m* through , where the assumed fractal dimension *d*_f_ was set to 2.41. It has been shown that the exact value of *d*_f_, in particular matching it to the actual fractal dimension of the final structure, has a very minor effect on the resulting structure.^48^ No rearrangement of particles due to bond breakage or rotational rearrangement was included. Such effects are, however, often regarded as secondary compared to the diffusivity and the sticking probability.^46^ Gravitational effects and rotational diffusion were also disregarded in this study.

### Methods for Comparing the Structures

#### Spatial Summary Statistics

This section introduces four summary statistics that were used to compare the experimental data and the simulated structures by considering the collections of centroids of the particles as spatial point patterns, which are realizations of a point process. A thorough description of point processes can be found in the literature.^49−51^ The summary statistics used in this study are the empty space function, *L*-function, clustering function, and mean cluster size function. All these summary statistics can be estimated from the observations of a point process Φ observed in the box *W* in . The underlying point process Φ is assumed to be stationary and isotropic, i.e., translation and rotation invariant.

The empty space function *F*: [0, ∞) → [0, 1] gives the probability that the distance from an arbitrary test point  to its nearest neighbor in Φ is less than or equal to *r* ≥ 0. Let *b*(*o*, *r*) be the ball of radius *r* ≥ 0 centered at  and *d*(*o*, Φ) be the shortest distance between *o* and points of the process. The empty space function is then given as
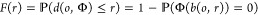
5where Φ(*b*(*o*, *r*)) denotes the number of points of the process in *b*(*o*, *r*). The longer it takes for the *F*-function to approach 1, the more empty space there is in the point pattern. An estimator for the empty space function is given in the Supporting Information.

A commonly used second-order characteristic is Ripley’s *K*-function *K*: [0, ∞) → [0, ∞), which measures the average number of points of the process that can be found within a distance *r* ≥ 0 from an arbitrary point *o* of the process Φ. If λ denotes the intensity, i.e., the mean number of points per unit volume, the *K*-function is defined as
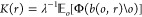
6where  denotes the conditional expectation given that there is a point of the process in *o*. Instead of using the *K*-function directly, a variance stabilized transformation, the *L*-function *L*: [0, ∞) → [0, ∞) was applied
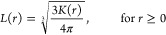
7Positive values of the centered function *L*(*r*) – *r* indicate clustering and negative values regularity. An estimator for the *K*-function can be found in the Supporting Information.

The clustering function *c*: [0, ∞) → [0, 1] is a third-order characteristic based on graph theory which has been extended to point processes.^52^ In the clustering function, one considers triplets of points where all of the points are within some distance *r* and compares it to the maximal theoretical number of such triplets. As such, the summary statistic can be interpreted as a measure of the internal connectivity around an arbitrary point at a distance *r*. For *o* ∈ Φ, the number of triplets within a distance *r* from *o* is given by
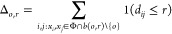
8The theoretical number of possible triplets for *o* ∈ Φ within a distance *r* is obtained as
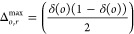
9where
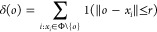
10The observed and theoretical numbers of triplets are compared through
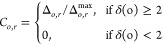
11and then, the expected value  is taken as a summary statistic. Values close to 1 are an indication of dense clusters. An estimator for the clustering function is given in the Supporting Information.

The mean cluster size function *M*: [0, ∞) → [0, ∞) is a summary statistic that describes how dense and spread out the clusters are.^35^ A geometric graph with the points *x* in Φ ∩ *W* as nodes and with connections between points *x*_*i*_ and *x*_*j*_ for which *d*_*ij*_ ≤ *r* is created. A cluster at the distance *r* can then be defined as the set of all points that are connected by such edges. Assume that there are *K* clusters for distance *r* and that cluster *k* has *n*_*k*_ points in it. The size of cluster *k* can then be measured by using the diameter of gyration
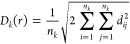
12The mean cluster size function in three dimensions for distance *r* was defined as
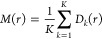
13for *r* ≥ 0. In order to account for edge effects, the so-called minus-sampling scheme was applied after the cluster construction, meaning that *M*(*r*) was calculated from points in the observation box at a distance further than *r* from the borders of the box.

#### Fractal Dimension

Clusters formed through DLCA and RLCA have been found to have fractal scaling properties, in the sense that the mass of an aggregate *m*_cl_ scales with the radius of gyration *R*_g_ according to
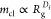
14where *D*_f_ is the fractal dimension. The fractal dimension *D*_f_ is often used as a fundamental description of the morphology of an aggregate. It provides a quantitative measure of the degree to which a structure fills the physical space.^53^ A fractal dimension of 1 corresponds to a line, 2 corresponds to a plane, and 3 corresponds to the whole space. In this project, the fractal dimensions of the simulated and experimental structures were estimated using the so-called box-counting algorithm and used as a further metric to compare the structural resemblance.^54^

#### Mass Transport

The mass transport through the gel structures was also used to compare the simulated structures to the experimental data as these properties describe the functionality of the gel in many applications. Here, we concentrate on two properties, flow and diffusion.

The flow of a fluid through a porous material may be driven by a pressure gradient or an external force, such as gravity. Assuming a steady creeping flow, the average flow velocity  in the direction γ (*x*, *y*, or *z*) through the material is described by Darcy’s law
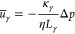
15where Δ*p* is the pressure drop driving the flow in the direction γ, *L*_γ_ is the material thickness, and η is the dynamic viscosity of the fluid.^55^ The permeability κ_γ_ is a characteristic property of the material, which describes how easily the fluid passes through the material in that direction. Permeability is related to the porosity of the material but is also affected by the shapes of the pores and their connectivity. The higher the permeability, the easier it is for the fluid to flow through the material structure.

The permeability can be computed from the three-dimensional pore structure of a material by solving the Navier–Stokes equations in the pore space, with an applied pressure drop or driving force to drive the flow, and computing the average velocity *u̅* from the numerical solution. Here, the Navier–Stokes equations were solved using the lattice Boltzmann method with the in-house software Gesualdo in a periodic simulated geometry with applied body force *F*. The permeability was then computed from (15) with *F* replacing the pressure gradient Δ*p*/*L*. Nonslip boundary conditions were used at the solid boundaries.

Diffusion is the migration or movement of particles due to random motion driven by the thermal energy measured by the diffusion coefficient. The higher the diffusion coefficient, the faster the movement. Diffusion through the gel structures was simulated in Gesualdo software by solving the diffusion equation
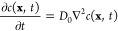
16where *c*(**x**, *t*) is the concentration which depends on location  and time *t* ∈ [0, ∞), and *D*_0_ is the free diffusion coefficient.^56^ A constant concentration difference *c*_2_ – *c*_1_ was applied over the structure in the direction γ (*x*, *y*, or *z*), and Neumann (zero normal flux) boundary conditions were used at the solid boundaries. At steady state, the effective diffusion coefficient *D*_eff,γ_ was then computed from Fick’s first law
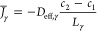
17where *L*_γ_ is again the thickness of the material and  is the average flux in the direction of the concentration gradient. The effective diffusion coefficient *D*_eff,γ_ is proportional to the free diffusion coefficient *D*_0_, and in order to get a quantity that only depends on the geometry of the aggregate, we calculate
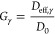
18which we call the geometry factor.^57^

### Parameter Selection

#### Parameter Values

The parameters in the sticking probability models were tuned by using a grid search approach. Using more advanced optimization methods was not feasible due to long simulation times. This limitation arose from the necessity to investigate small sticking probabilities and acquire structures large enough to reliably estimate different characteristics of the material.

As in the earlier studies,^35,58^ simulations with a constant sticking probability were performed with the values
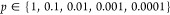
For the mass-dependent sticking probability, simulations were carried out with all combinations of
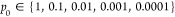

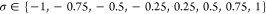
Here, *p*_0_ is the sticking probability for single particles with average mass and σ decides how this probability changes with the cluster size. Simulations with the neighbor-dependent sticking probability were performed with all combinations of
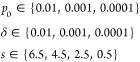
with *s* given in nm. The parameter *p*_0_ is the probability of aggregation when no other particles are present, while δ is the increase in probability for every neighbor within surface-to-surface distance *s* + *r*_*i*_ + *r*_*j*_ to the colliding particles. Here, *r*_*i*_ and *r*_*j*_ denote the radii of the particles that the distance is calculated between. Out of all of the simulations, the smallest value of the evaluation metric *S* (see below) was obtained for the neighbor-dependent sticking probability with parameters *p*_0_ = 0.0001, δ = 0.001, and *s* = 4.5 nm. In order to improve the goodness-of-fit further, a more local grid search was performed with the parameters
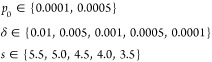


#### Selection of the Best Parameters

The best set of parameters for each of the models for the sticking probability was selected based on the spatial summary statistics. The summary statistics were calculated for *r* in the range 0–50 nm in R using the **spatstat** and **SGCS** packages.^59,60^ To achieve more robust results, the experimental and simulated data were divided into subpatterns, for which the summary statistics were calculated and then averaged over. The real data were divided into four subpatterns of size 360 × 520 × 100 nm^3^, whereas the simulated data were divided into six subpatterns of the same size. These subpatterns have enough space between them to be considered to be approximately independent replicates. The average over subpatterns was calculated by pooling according to
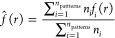
19where *n*_patterns_ is the number of subpatterns, *f*_*i*_(*r*) is the summary statistic for subpattern *i* at distance *r*, and *n*_*i*_ is the number of points in subpattern *i*.

The pooled summary statistics from the simulated and STEM data were compared using an evaluation metric *S* of the form
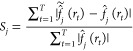
20where *j* refers to one of the summary statistics *F*, *L*, or *c*, and  and  are the pooled summary statistics from the experimental and simulated data, respectively. The mean cluster size function *M* was not included in the evaluation metric since it was found to have a high variance. The full evaluation metric *S* was thus given by
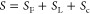
21

## Results

### Goodness-of-Fit Based on the Summary Statistics

**Figure 4:**
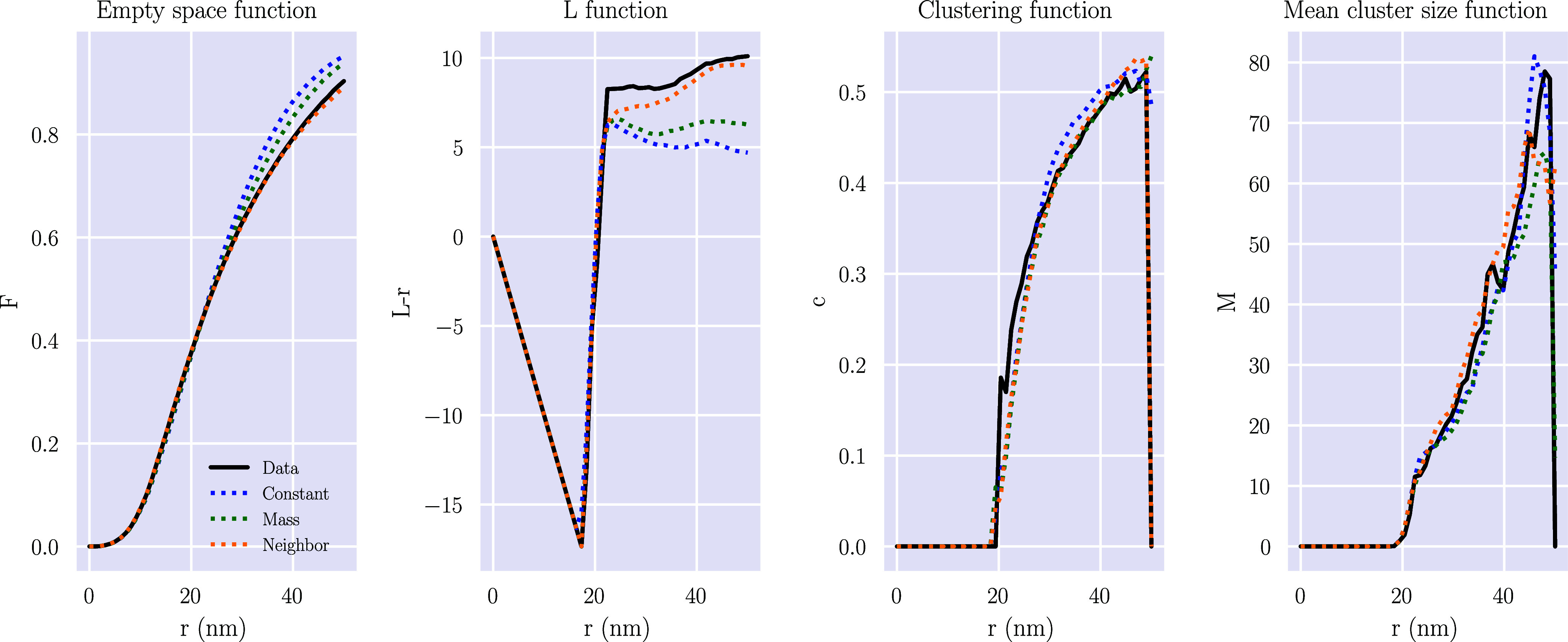
Summary statistics for the experimental data and the simulations with each of the sticking probability models that resulted in the best goodness-of-fit (parameter values given in Table 1). Note that the centered *L*(*r*) – *r* is plotted for the *L*-function.

The parameters that resulted in the best goodness-of-fit for the summary statistics can be found in Table 1, and the summary statistics from these simulations and for the experimental data are plotted in Figure 4.

**Table 1:** Parameter Values That Minimized the Evaluation Metric *S* in eq 21 for the Three Sticking Probability Models

model	parameters	*S*
constant	*p* = 10^–4^	0.5315
mass	*p*_0_ = 10^–4^, σ = 0.5	0.3874
neighbor	*p*_0_ = 10^–4^, δ = 5 × 10^–4^, *s* = 4.0	0.1585

For all of the models, relatively small probabilities in the RLCA regime gave the best results in terms of the summary statistics. Hence, there seems to be strong repulsion between the particles. For the mass model, the best value of σ was obtained to be positive, meaning that the sticking probability increases as clusters grow larger. From the simulations of the neighbor model, the best value of *s* was 4.0 nm. For average sized particles, this means that neighbors within a surface-to-surface distance up to approximately 25 nm contributed to the sticking probability since the mean radius of the particles is about 10.5 nm.

The empty space function *F* of the neighbor model is very close to the *F*-function estimated from the data. The corresponding curves for the mass model and constant probability model are above the data curve, and the curve for the neighbor model indicates that patterns based on these two models have less empty space and less dense clusters. Results based on the centered *L*-function are very similar. The *L*-function of the neighbor model follows the data curve well while the *L*-functions for the constant and mass models lie further below the two other curves indicating less dense clustering. However, the neighbor and mass models describe the data equally well and are slightly better than the constant model measured by the clustering function *c*. For the mean cluster size function *M*, all models describe the data fairly well.

**Figure 5:**
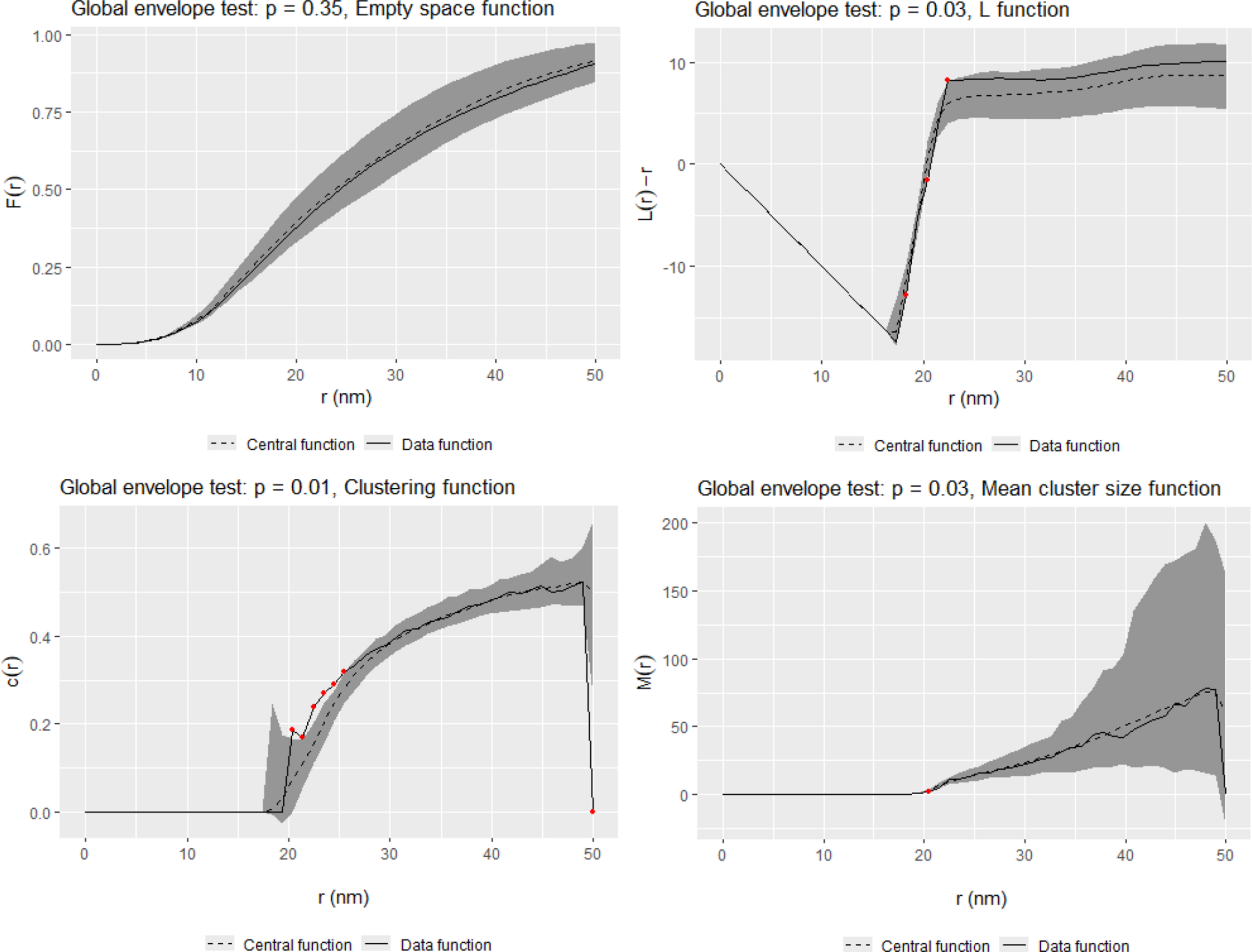
Global envelope tests from 99 simulations using the neighbor model with parameters *p*_0_ = 10^–4^, σ = 5 × 10^–4^, and *s* = 4.0 nm. Here, *p* denotes the *p*-values of the tests, which were performed separately for the different summary statistics.

We evaluated the goodness-of-fit of the best-fitting neighbor model (with parameter values on the last row in Table 1) by using global envelope tests.^61^ In a global envelope test, one tests if a certain null model is appropriate to describe an observed point pattern based on performing Monte Carlo simulations from the chosen null model and then comparing summary statistics from the simulated and observed patterns. Envelopes are calculated for the summary statistics from simulations of the null model, and the null model is rejected if the observed summary statistic is not entirely within the envelope. The test also produces a *p*-value at the desired significance level, α, and the null model is rejected if *p* ≤ α. Details on the test that was used can be found in the Supporting Information. As shown in Figure 5, the data curve of the *F*-function is entirely inside the envelope, whereas for the other summary statistics, the null model is rejected using significance level α = 0.05.

### Fractal Dimension

The fractal dimension was estimated using the box-counting algorithm for the structures used for Table 1. As shown in Table 2, the fractal dimensions are similar considering the standard errors from the linear regression in the box-counting algorithm. However, as in the case of the summary statistics, a constant sticking probability gives a fractal dimension that is the furthest from the experimental data, while the neighbor model performs best.

**Table 2:** Estimated Fractal Dimensions *D*_f_ with Standard Error (SE) from Linear Regression

	*D*_f_ ± SE
data	2.3807 ± 0.0109
constant	2.3426 ± 0.0160
mass	2.3540 ± 0.0136
neighbor	2.3933 ± 0.0102

### Mass Transport

Flow and diffusion were simulated for 10 structures generated from each of the different sticking probability models, using the same parameters that resulted in the best goodness-of-fit of the spatial summary statistics; see Table 1. Structures were generated with the same box dimensions as the experimental data (740 × 1075 × 100 nm^3^) in order to enable a simple comparison. Since the experimental sample is very thin in the *z*-direction, mass transport simulations were only performed in the *x*- and *y*-directions.

**Figure 6:**
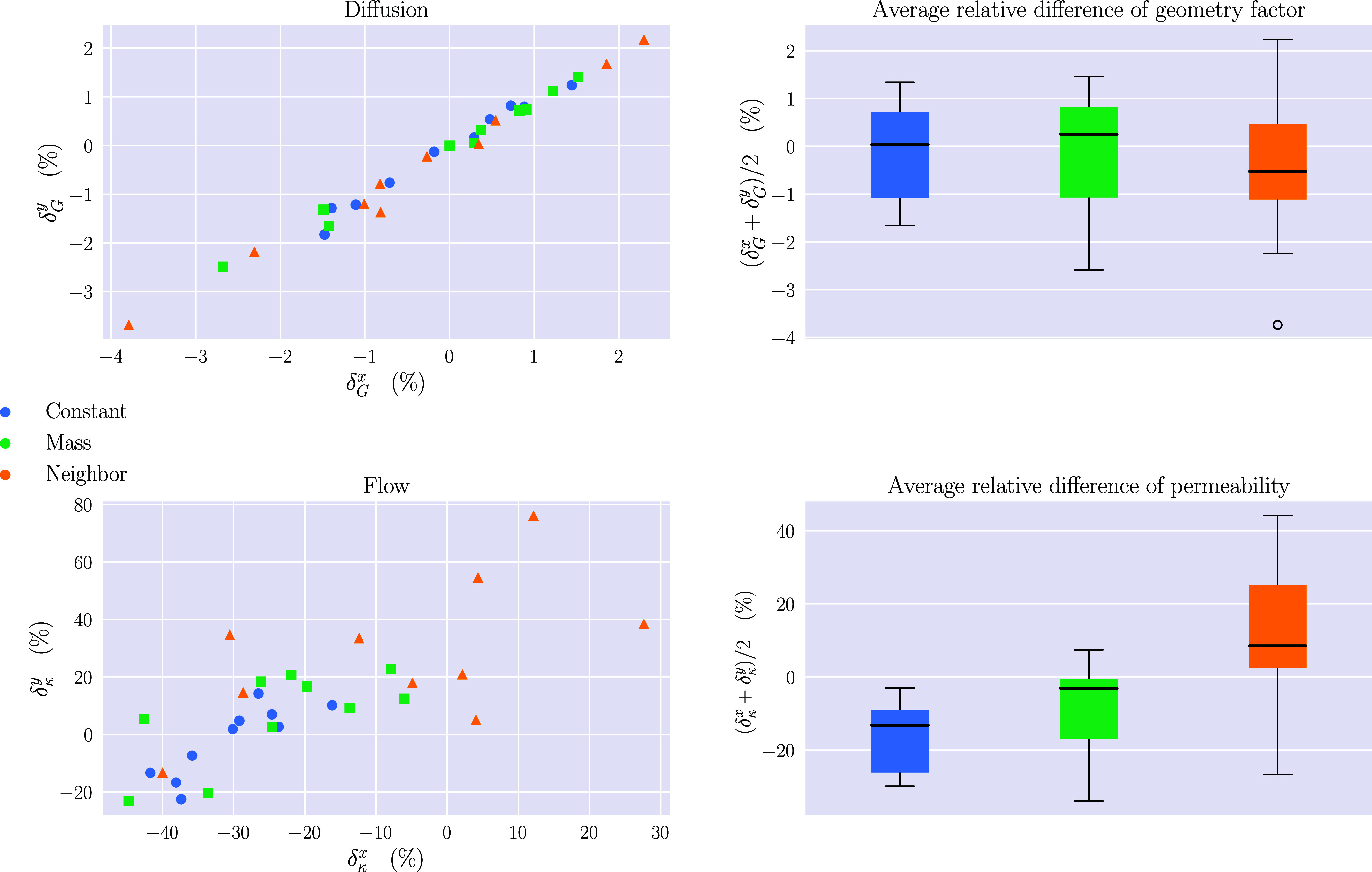
Relative differences δ between the simulated structures and the experimental values of the geometry coefficient *G* (top) and permeability κ (bottom). Simulations were carried out in two directions *x* and *y*. For the physical gel, κ was obtained to be approximately 2.160 × 10^–16^ m^2^ in the *x*-direction and 1.569 × 10^–16^ m^2^ in the *y*-direction. The corresponding values for the geometry factor were 0.845 (unitless) and 0.846 (unitless). In the scatter plots, each point corresponds to one of the generated structures and shows the relative difference compared to the physical gel in both simulation directions. The boxplots show the average over the two directions for each structure.

The relative difference δ between the values of the flow permeability κ_γ_ and the diffusion geometry factor *G*_γ_ = *D*_eff,γ_/*D*_0_ obtained from the generated structures and the corresponding experimental values were calculated in each direction of simulation. For instance, the relative difference of the diffusion coefficient when simulating mass transport in the *x*-direction was calculated as . Here, *G*_*x*,sim_ represents the geometry factor in the *x*-direction of a simulated structure, and *G*_*x*,exp_ represents the geometry factor in the *x*-direction of the experimental structure. Therefore, the relative difference should have a value of 0 if the simulations and experimental results agree. Note, however, that the experimental structure represents only a small sample from the material, and a large variability in geometry factors and permeabilities would likely be observed if more experimental data were obtained. The obtained results for κ and *G* can be seen in Figure 6.

Overall, the structures with constant sticking probability deviate most from the experimental data. The mass and neighbor models fit the experimental data equally well, considering the range of permeabilities from the simulations. In terms of the geometry factor, all three models fit the data well, and there is not much difference between simulations in the different directions.

## Discussion

The results based on the summary statistics, fractal dimension, and mass transport properties clearly indicate that both the mass model and the neighbor model fit the experimental data better than the constant probability model. Furthermore, the overall fit of the neighbor model to the STEM data seems better than the fit of the mass model. The rather dense clusters of the data and the amount of empty space are well captured by the neighbor model, which makes the particles stick more likely to positions with many nearby particles.

The goodness-of-fit of the models was first investigated using the four summary statistics, namely, the empty space function, centered *L*-function, and clustering function, which were also used in the parameter estimation, and in addition, the mean cluster size function. The mean cluster size function was not included in the evaluation metric *S* minimized in the parameter estimation since at large distances, it has to be computed based on only a few points due to the minus-sampling edge correction scheme, giving rise to increased variation.

While the empty space function and the centered *L*-function clearly chose the neighbor model as the best-fitting model, both models seem to fit equally well according to the clustering function *c* and mean cluster size function *M*. In terms of the clustering function, neither of the models fits the data very well at particle center distances *r* in the range 20–27 nm. These values of *r* are approximately the same as the diameter of most of the particles in the simulation, meaning that only the nearest neighbors will affect the values of the *c*—function at such short distances. The systematic deviations may therefore be due to the differences in the fitted particle size distribution and the nearest neighbor distances in the experimental data.

The goodness-of-fit of the best-fitting model, i.e., the neighbor model with parameter values given on the last row in Table 1, was tested by global envelope tests, where empirical summary functions were compared to the envelopes based on simulations of the model. According to the empty space function *F*, the model fits very well to the STEM data, but some deviations of the experimental curve and the envelopes occur for the other three summary statistics. However, all of the empirical curves are outside the envelopes at particle center distances *r* in the range 18–26 nm, at which the particle diameters have a large impact on the values of these three summary statistics. The point outside the envelope at 50 nm for the clustering function is not relevant since the minus-sampling scheme is applied and the experimental structure is only 100 nm in one direction. Overall, the null neighbor model seems to describe the observed structure rather well even though the data curves are not completely inside the envelopes.

The goodness-of-fit of the models was also investigated in terms of the fractal dimensions and mass transport properties for flow and diffusion. The fractal dimension computed from the neighbor model simulations was closest to that for the STEM data. For the constant probability and mass models, the fractal dimension was slightly lower, indicating less complex shapes of clusters compared to the data. The fractal dimension computed here is higher than the one computed from the radial distribution function in previous work^32,34^ (around 2.4 compared to 2.2 for RLCA structures with ϕ = 0.104). This may be due to the different methods used, where the box counting method used here also takes the extent of the particles into account. Furthermore, the fractal dimension was computed here in a rather thin slice. The fractal dimension should therefore mostly be thought of as a way of comparing the different generated structures and experimental data.

The average geometry factor related to the diffusion coefficient lies between the first and third quartiles for each model, and therefore, the geometry factors of the three models are very similar. This is reasonable since the geometry factor mainly depends on the volume fraction accessible to diffusion and is not very sensitive to finer details of the geometry. Since the concentration of primary particles used in the simulations was estimated from the experimental data, the volume fractions were similar for the experimental and simulated materials.

The permeability, on the other hand, depends on the width and connectivity of the wider channels in the structure through which the fluid can pass. The permeability results for constant aggregation probability (average κ = 1.52 × 10^–16^ m^2^) are in agreement with results in the literature for RLCA structures with constant particle diameter,^34^ assuming a volume fraction ϕ = 0.104 and diameter of 21 nm. The particle size distribution thus has little effect on the permeability on average while resulting in a greater variability between simulations. Given that the neighbor model gives rise to denser clustering of particles and smaller values of the empty space function than the constant probability and mass models, as seen in the summary statistics, it is reasonable that the structures constructed using the neighbor model also have higher average flow permeability, as seen in Figure 6. However, it is difficult to determine whether the neighbor model or the mass model would give the permeability closest to the data. Since only 10 structures were generated and only one experimental data set of a rather small volume was available, it seems likely that either model could result in structures with similar permeability as in the data.

While DLVO theory implemented in discrete element models or Langevin dynamics simulations may be used to simulate gel structures similar to the ones studied here, previous studies indicate that the gel structures are not well reproduced.^11,20^ This failure may be due to the inaccurate modeling of the interaction between larger clusters of particles and the small-sized particles, or entropy effects caused by hydrodynamic interaction with water molecules.^62^ Our results also indicate that interactions involving several particles are of importance for the final gel structure. This suggests that the DLVO potentials would need to be modified to model silica gel aggregation accurately. Furthermore, a more realistic simulation of the dynamics of gel aggregation using these methods may be more costly computationally than the RLCA models used here. To simulate an RLCA model using a single processor with the lowest aggregation probability 10^–4^ took approximately 6 h in our setup, while the model with nonconstant probability resulting in the best-fitting summary statistics took only about 1 h.

The standard DLCA and RLCA models (which model the complete dynamics) were also studied in our earlier work^35^ and correspond to the constant probability models here. The only differences between the models are that the constant particle diameter^35^ was replaced by a distribution of diameters in this work and that the solid volume fraction was adjusted. The conclusion that the lower aggregation probabilities 10^–3^ and 10^–4^ work better than the larger ones remained. Some Gibbs point process models with rather simple potential functions were also suggested for the final silica gel structure.^35^ A similar approach has even been suggested for alumina-supported iron nanoparticles extracted from environmental transmission electron microscopy images.^63^ The goodness-of-fit of these models measured by the same spatial summary statistics as used here was better than the goodness-of-fit of the DLCA and RLCA models and comparable to the goodness-of-fit of the mass and neighbor models suggested in this paper. However, the Gibbs models are static, not dynamic, and are suitable only to model the final structure of the gel and not the entire dynamics of the gel formation.

In our work, particles of radius around 10 nm in a gel with pH 7.8 were studied. The DLCA models have been used to study the structural and mechanical properties of silica aerogels with smaller particles of radius 2–4 nm in an experimentally synthesized wet gel with a pH of 7.0^36^. In that study, the fractal dimension increases slightly with increasing relative density of the silica aerogel network but is not affected by the particle size. Furthermore, the fractal dimension of the gel was determined to be 2.44, agreeing well with the corresponding DLCA-based computationally obtained value 2.51 ± 0.05. The authors concluded that the DLCA model seems to be able to produce structures with similar fractal dimension as in the data in this particular case. The fractal dimensions in our experiment were quite similar, 2.38 for the data, and 2.35 and 2.39 for the mass and neighbor models, respectively. This further indicates that the fractal dimension is not much affected by the particle size. However, the mass and neighbor models with nonconstant aggregation probability introduced here seem to result in structures with fractal dimensions that are closer to the fractal dimension of the data than that in the structures obtained by the DLCA model. Furthermore, Abdusalamov et al.^36^ did not present any detailed study of the spatial structure in terms of summary statistics, as was done in our study.

## Conclusions

The aim of this work was to develop a dynamic model for aggregation that gives structures similar to the silica structures in hand and to provide a better understanding of the aggregation dynamics of colloidal silica. The DLVO theory can provide physically reasonable models for the colloidal gels but describes only the interaction between two spherical particles and not between larger clusters. Therefore, the structure of silica is not well predicted by these models.^11,20^

Furthermore, neither the standard DLCA model nor the RLCA model seems to be fully satisfactory to model the aggregation of silica particles^35^ which may be due to the constant sticking probability. It was suggested^35^ that allowing the particle size vary instead of it being constant could make a difference. However, this change, which we made in this study, does not improve the performance of the models much. Therefore, we suggested cluster aggregation models where the sticking probability depends on the masses of colliding clusters or on the number of particles close to the collision site and therefore varies as the aggregation process advances.

We compared the structures produced by three sticking probability models, constant probability, mass, and neighbor models, to the observed silica structure using several measures. First, summary statistics from the spatial point process theory were used to compare the spatial patterns. Second, the fractal dimensions with higher values having the tendency to be connected to more complicated cluster shapes were compared. Finally, the mass transport properties were compared by simulating the flow and diffusion through the structures. The neighbor and mass models with varying sticking probabilities performed clearly better than the constant probability model. Based on all of the goodness-of-fit results above, we can conclude that the neighbor model, where the sticking probability depends on the number of particles near the collision site, describes the STEM data fairly well. It works better than the mass model, and both the neighbor and mass models are clearly better than the constant probability model. However, the exact parameter values may depend on the sample and the conditions under which the sample has been prepared. The results of this study suggest that the interaction with multiple particles at the collision site is an important feature in describing the aggregation dynamics under the conditions under which the experimental gel was created. The model can be tuned to different numbers of particles, their size, and their shape in order to deal with a large variety of systems. However, some other experimental conditions would lead to slightly different structures, and therefore, the optimal parameter values would most likely be different. Our main lesson is that when modeling the aggregation process by cluster aggregation models similar to DLCA and RLCA, it may be useful to let the aggregation probability vary in time instead of being constant. Here, we have suggested two such models and demonstrated that these new models describe the observed silica structures well, better than using a constant value for the probability. Therefore, further testing of the parameters and replicated experiments would be necessary to draw some stronger conclusions.

An interesting topic for future research would be to investigate whether the models based on the DLVO theory could be generalized so that they would include interaction between clusters of particles, which would make them more suitable for silica gel formation. The spatial summary statistics used here could again be applied to estimate the goodness of fit. Also, the models suggested here could further benefit from the knowledge gained from local particle–particle interaction models included in DLVO theory or molecular dynamics models. Finally, it would be interesting to fit our models to gels produced under different conditions, optimize the parameters, and see how much the parameter values are influenced by the gelation conditions.

## Supporting Information Available

Additional details on methods for estimation of the spatial summary statistics (PDF)
