# Supplementary material for: Modeling Colloidal Particle Aggregation Using Cluster Aggregation with Multiple Particle Interactions
Source: J Phys Chem B. 2024 Apr 30;128(18):4513–24. doi: 10.1021/acs.jpcb.3c07992 (PMC11089502; doi:10.1021/acs.jpcb.3c07992)
Supplement: Supplementary file 1 — jp3c07992_si_001.pdf [file jp3c07992_si_001.pdf]

Supporting information: Modeling Colloidal Particle Aggregation using Cluster Aggregation with Multiple Particle Interaction

Jakob Antonsson,[†] Charlotte Hamngren Blomqvist[¶,‡] Eva Olsson,[‡] Tobias Gebäck,[*,†] and Aila Särkkä[†]

†*Department of Mathematical Sciences, Chalmers University of Technology and University of Gothenburg, SE-412 96 Gothenburg, Sweden*

‡*Department of Physics, Chalmers University of Technology, SE-412 96 Gothenburg, Sweden*

E-mail: tobias.geback@chalmers.se

Estimation of spatial summary statistics

Empty space function

The empty space function was estimated using an unbiased Kaplan-Meier estimator with the hazard rate

$$\hat{h}(r) = \frac{\nu_2(\partial(\Phi_{\oplus r}) \cap W_{\ominus r})}{\nu_3(W_{\ominus r} \backslash \Phi_{\oplus r})} \tag{1}$$

for $\Phi_{\oplus r} = \Phi \oplus b(o, r)$ where $\oplus$ is the Minkowski addition, $W_{\ominus r} = W \ominus b(o, r)$ where $\ominus$ is Minkowski subtraction[1]. Here, $\nu_2(A)$ and $\nu_3(A)$ denotes the area and the volume of a set

$A \in \mathbb{R}^3$, respectively. The full estimator for $F(r)$ is then given by

$$\hat{F}(r) = 1 - \exp\left[- \int_0^r \hat{h}(s) ds \right]. \tag{2}$$

L-function

The $L-$function is based on the $K-$function which was estimated as[2]

$$\hat{K}(r) = \frac{\hat{\lambda}^{-1}}{n} \sum_{i=1}^{n} \sum_{j \neq i} w_{ij}^{-1} \mathbb{1}(d_{ij} \leq r) \tag{3}$$

where

$$w_{ij} = w(x_i, d_{ij}) = \frac{\nu_2(\partial(b(x_i, d_{ij}) \cap W))}{\nu_2(\partial b(x_i, d_{ij}))}, \tag{4}$$

and

$$\hat{\lambda} = \frac{n-1}{\nu_3(W)}. \tag{5}$$

In analogy with the L-function itself, an estimator $\hat{L}$ for the L-function is obtained from $\hat{K}$ by

$$\hat{L}(r) = \sqrt[3]{\frac{3\hat{K}(r)}{4\pi}}, \quad \text{for } r \geq 0. \tag{6}$$

Clustering function

The clustering function $c(r)$ was estimated as $\tilde{c}(r) = \mathbb{E}_o[\Delta_{o,r}]/\mathbb{E}_o[\Delta_{o,r}^{\max}]$ by using that[3]

$$\mathbb{E}_o[\Delta_{o,r}] \approx \sum_{i: x_i \in \Phi \cap W} \sum_{\substack{j,k: x_j, x_k \in \\ \Phi \cap W \cap b(x_i, r) \setminus \{x_i\}}} \frac{\mathbb{1}(||x_j - x_k|| < r)}{n} \tag{7}$$

and

$$\mathbb{E}_o[\Delta_{o,r}^{\max}] \approx \sum_{i: x_i \in \Phi \cap W} \frac{1}{2n} (\delta(x_i)^2 - \delta(x_i)). \tag{8}$$

Edge effects were handled by applying the so-called minus-sampling scheme.

Global envelope tests

Let $T_{\text{obs}}(r)$ be the value of a summary statistic estimated from the observed data at distance $r \geq 0$. An *envelope* is a band bounded by the functions $T_{\text{low}}(r)$ and $T_{\text{upp}}(r)$ on an interval I[4]. A global envelope test is a statistical test that rejects the null hypothesis H_0, i.e., that the null model is appropriate for the data, in the case that the observed function $T_{\text{obs}}(r)$ is not completely inside the envelope

$$\varphi_{\text{env}}(T_{\text{obs}}) = \mathbb{1}(\exists r \in I : T_{\text{obs}}(r) \notin (T_{\text{low}}(r), T_{\text{upp}}(r))). \tag{9}$$

The bounds are established in such a way that the test has a controlled global type I probability for a certain number of simulations n_{sim}. In this study, the *directional quantile* MAD envelope test was used[4]. In addition to only requiring a small number of simulations, this test considers variations of the variance for the summary statistic for different values of r as well as asymmetry in the distribution.

Present address: ¶C.H.B.: Department of Physics, University of Gothenburg, Box 100, SE-405 30 Gothenburg, Sweden

References

(1) Baddeley, A.; Gill, R. D. Kaplan-Meier Estimators of Distance Distributions for Spatial Point Processes. *The Annals of Statistics* **1997**, *25*, 263–292.

(2) Diggle, P. J. *Statistical Analysis of Spatial and Spatio-Temporal Point Patterns.*; Chapman and Hall/CRC, Boca Raton, 2014; Vol. 3rd ed.

(3) Rajala, T. *Spatial point processes and graph based statistical features*; 2010; Available at http://www.math.jyu.fi/research/pspdf/385.pdf, accessed Aug 18, 2023.

(4) Myllymäki, M.; Mrkvička, T.; Grabarnik, P.; Seijo, H.; Hahn, U. Global envelope tests for spatial processes. *Journal of the Royal Statistical Society: Series B (Statistical Methodology)* **2016**, *79*, 381–404.
